# Sustained elevation of Epstein–Barr virus antibody levels preceding clinical onset of nasopharyngeal carcinoma

**DOI:** 10.1038/sj.bjc.6603609

**Published:** 2007-02-06

**Authors:** M F Ji, D K Wang, Y L Yu, Y Q Guo, J S Liang, W M Cheng, Y S Zong, K H Chan, S P Ng, W I Wei, D T T Chua, J S T Sham, M H Ng

**Affiliations:** 1 Cancer Research Institute of Zhongshan City, Zhongshan City, PRC; 2 Department of Pathology, Sun Yat Sen Medical College, Sun Yat Sen University, Guangzhou, PRC; 3 Department of Microbiology, Hong Kong SAR, PRC; 4 Department of Surgery, Hong Kong SAR, PRC; 5 Department of Clinical Oncology, Li Ka Shing Faculty of Medicine, The University of Hong Kong, Hong Kong SAR, PRC

**Keywords:** NPC, EBV, serology, cancer screening, tumour marker

## Abstract

We have monitored Epstein–Barr virus (EBV) IgA antibody levels of 39 nasopharyngeal carcinoma (NPC) cases for up to 15 years before clinical onset of NPC, and assessed preclinical serologic status of another 68 cases. Our results identify a serologic window preceding diagnosis when antibody levels are raised and sustained. This window can persist for as long as 10 years, with a mean duration estimated to as 37±28 months. Ninety-seven of these 107 NPC cases exhibited such a window. Cases that did not may reflect individual antibody response to EBV. Serologic screening at enrollment identified those cases who had already entered the window and became clinically manifested earlier (median=28 months) than those who entered the window after enrollment (median=90 months). The former account for 19 of 21 cases diagnosed within 2 years of screening. Nasopharyngeal carcinoma risk levels among seropositive subjects were also highest during this period. Both prediction rates and risk levels declined thereafter; cases detected at later times were composed of increasing proportions of individuals who entered the serological window after screening. Our findings establish EBV antibody as an early marker of NPC and suggest that repeated screening to monitor cases as they enter this window has considerable predictive value, with practical consequences for cancer treatment.

Nasopharyngeal carcinoma (NPC) is a major tumour in China and elsewhere in Asia, as well as in restricted areas of Africa (Yu and Yuan, 2002). It has a strong association with the human herpesvirus, Epstein–Barr virus (EBV) (Raab-Traub, 2002), with general elevation of serum EBV antibody level being an outstanding feature of the tumour (Olds *et al*, 1966; Henle *et al*, 1970; Henle and Henle, 1976; Ho *et al*, 1978a; Zeng *et al*, 1983; Cheng *et al*, 2002; Chan *et al*, 2003; Fachiroh *et al*, 2004). EBV antibody screening in sera of populations in high incidence areas for NPC can be useful in predicting onset of the cancer (Yi *et al*, 1980; Zeng *et al*, 1983; Chien *et al*, 2001; Ji *et al*, 2003a, 2003b). This is presumed to reflect rises in antibody levels at an early stage in cancer development (Ho *et al*, 1978a; Cheng *et al*, 2002).

To investigate the relationship between the serologic changes and disease progression, we have conducted a prospective study in a high-incidence area in southern China. The study has extended over 16 years (between 1986 and 2002) and has involved 42 048 adult subjects (Ji *et al*, 2003b). Recruitment extended over 18 months, when each study subject was given a serologic test and a clinical examination. Seropositive subjects identified as having high-serum IgA antibody against EB viral capsid antigen (VCA) at enrollment to the study were clinically and serologically followed up on eight subsequent occasions between years 3 and 13. Some seronegative subjects, having low IgA VCA at the time of enrollment, were similarly examined on these occasions. Nasopharyngeal carcinoma cases were detected either by clinical examination conducted during follow-up, when they symptomatically presented to the out patients department (OPD) of our hospital, or could be traced to other hospitals. We found that cumulative NPC incidence was 20 times higher for the seropositive subjects than seronegative subjects (Ji *et al*, 2003b).

We describe here changes in the preclinical serologic status observed during the intervals between enrollment and clinical manifestation of the tumour. The results reveal a window of about 3 years immediately preceding clinical onset, when the antibody level was elevated and maintained at high levels. It was further shown that virtually all cases entered this serologic window. Those who had already entered the window at the time of enrollment were at a more advanced stage of preclinical tumour development than those who entered the window only after enrollment.

## MATERIALS AND METHODS

### Recruitment

We have conducted a clinical and serologic follow-up study of NPC extending over a period of 16 years between December 1986 and December 2002 in Zhongshan City in Southern China. The study area has one of the highest incidences of the cancer in the world (Yu and Yuan, 2002). Forty-two thousand and forty-eight normal adults, 19 325 male and 22 723 females, aged 30–59 years (mean age=40.9 years), were recruited to the study with informed consent. Recruitment extended over a period of 18 months between December 1986 and June 1988, and the study subjects were followed up until December 2002. A serum sample was taken from each subject at enrollment for EBV serology, and each study subject was given a routine clinical examination, including examination of the nasopharynx by endoscope or mirror. Biopsy was taken for histopathology where clinically indicated. Those with VCA IgA titre ⩾cutoff value at the time of enrollment were designated as belonging to the ‘seropositive group’ and those with VCA IgA titre < cutoff value to the ‘seronegative group’.

### Follow-up

We performed another 22 279 clinical examinations on eight subsequent occasions between year 3 and 13 of the study; 13 186 examinations were performed for the seropositive group and 9093 examination for the seronegative group and serum samples taken on each occasion for determination of EBV antibody levels.

### Serology

Epstein–Barr virus-specific VCA IgA antibody level was determined by titration using an immunoenzymic assay described previously (Yi *et al*, 1980). Serum samples collected during enrollment and on subsequent occasions were tested in separate batches. They were kept at 4^°^C and used within 1 month or kept at −20°C for longer periods. The cutoff titres were set at 1 : 5 to 1 : 40 on different occasions to achieve a comparable assay specificity of 90–95% (mean=92%) between different test batches. The corresponding sensitivity was 91–97%. Elevated antibody levels were indicated when VCA IgA titre ⩾cutoff value.

## RESULTS

### Nasopharyngeal carcinoma cases detected

Table 1 shows that serologic screening conducted at enrollment identified 3093 subjects with VCA IgA titre greater than or equal to their respective cutoff, and are referred hitherto as ‘seropositive’. The others had lower titre than the cutoff titre and are referred to as ‘seronegative’. Clinical examination, including an examination of the nasopharynx, was conducted for all the study subjects. It detected 40 overt cases, 39 from among the seropositive subjects.

A total of 13 186 follow-up examinations performed for the seropositive subjects during routine screening resulted in detection of an additional 34 cases of NPC. Twenty-two other cases were detected from this group not by routine screening on these occasions but by referral to our clinic after onset of symptoms related to underlying NPC. These symptoms included epistaxis, nasal obstruction, hearing impairment, neck lump, headache, facial numbness and double vision. Another 9093 examinations were performed during routine screening for the seronegative subjects during the same time, but the effort did not detect a single NPC case. Seventy-five NPC cases, however, were detected from this group but only after symptoms occurred, which prompted patients to seek medical advice in our hospital or other hospitals in the region.

In all, a total of 131 cases were detected from both cohorts 8 months to 15 years after enrollment, 56 from the seropositive group and 75 from the seronegative group. The overall prediction rate (56/131) achieved by serologic screening conducted at the time of enrollment is similar to that reported by Chien *et al* (2001), but lower than that achieved by Yi *et al* (1980) and Zeng *et al* (1983). The cumulative incidence of the entire study population calculated over 15 years is 21 per 10^5^ person-year. The cumulative NPC incidence of the seropositive group was 5.8 times higher than the entire study population and that of the seronegative group, 0.5 times lower.

### Diagnosis of NPC

**Figure 1:**
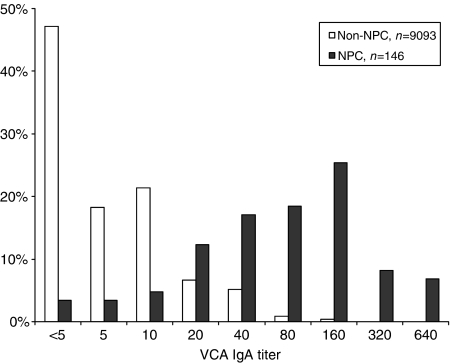
Serum VCA IgA titre of NPC and non-NPC. Serum samples were taken at times of diagnosis from 146 NPC patients (solid bar) and another 9093 samples were taken at different times during study from non-NPC subjects (open bar). Viral capsid antigen IgA titres of NPC patients are significantly higher than non-NPC subjects (*χ*^2^=2752.383, *P*<0.0001).

Review of histopathology showed that 166 of a total 171 cases detected among the study subjects were class II or III non-keratinizing carcinoma, according to World Health Organization (WHO) International Histological Classification of Tumors (Shanmugaratnam, 1991), and five cases were WHO class I keratinizing carcinoma. However, we did not also conduct *in situ* hybridization or immunohistochemistry to confirm the presence of EBV in the tumours. Nevertheless, serum samples were taken at times of diagnosis from 146 of these cases, and the results show that VCA IgA titres of these patients were markedly elevated compared with non-NPC subjects (Figure 1).

### Disease staging

All patients with newly diagnosed NPC underwent clinical staging workup, which included complete physical examination, endoscopy of nasopharynx, haematology and renal/liver biochemistry, chest X-ray and computed tomography of nasopharynx and neck. In this report, all cases of NPC were retrospectively staged according to the 1997 American Joint Committee of Cancer (AJCC)/International Union Against Cancer (UICC) TNM stage classification system. Early-stage disease was defined by AJCC/UICC stages I–II disease and advanced stage by AJCC/UICC stages III–IV disease. Briefly, stage I disease refers to disease confined to nasopharynx only, without cervical nodal involvement; stage II disease involves extension to parapharyngeal space, nasal fossa or oropharynx only, or presence of unilateral cervical nodes ⩽6 cm above the supraclavicular fossa; stage III disease has involvement of skull base or other paranasal sinus, or presence of bilateral cervical nodal disease ⩽6 cm above the supraclavicular fossa; stage IV disease includes intracranial extension, cranial neuropathy or nodal size >6 cm, or involving the supraclavicular fossa.

Seventy-four of the 171 cases were detected during routine screening, including 40 at enrollment and 34 during follow-up. The other 97 cases were detected only after the onset of symptoms related to underlying NPC, which occurred at different times during follow-up (Table 2). Cases being diagnosed after onset of symptoms referred only to those with symptoms that were clearly related to the underlying NPC, which prompted patients to seek medical advice leading to the diagnosis. Review of clinical records shows that only 19.6% of the cases diagnosed after the occurrence of symptoms had early stage disease (AJCC/UICC stages I–II), which is similar to the percentage of early-stage disease detected in 1629 NPC cases concurrently presenting to our OPDs between 1988 and 2003. By comparison, 67.6% of the cases detected by our screening program had early-stage NPC. This confirms the previous findings that the onset of most symptoms in NPC tend to occur at a relatively late stage of the disease. Thus, participation in the present study would not enhance the awareness of symptoms to such an extent as to facilitate early diagnosis of the cancer.

### Occurrence of NPC after enrollment

**Figure 2:**
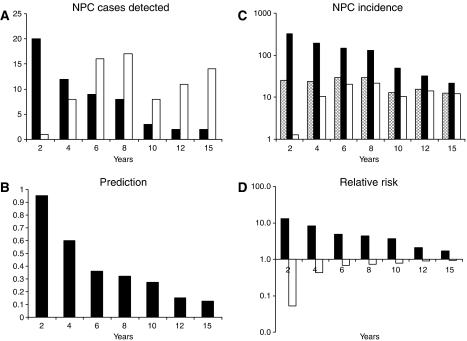
Occurrence of NPC after screening. A total of 131 NPC cases (hatched bar) were detected at different times from 8 months to 15 years after enrollment, 56 from seropositive group (solid bar) and 75 from seronegative group (open bar). Occurrence was analysed in successive 2 years in the first 12 years and the final 3 years in years 13–15 of the study; incidence is expressed in cases per 10^5^ person-year; prediction is calculated as percentage of cases detected from seropositive group; relative risk is calculated as ratio of incidence of seropositive or seronegative group to the corresponding value for the total population.

Figure 2A shows the occurrence of the 131 NPC cases as detected at 2-yearly intervals until year 12, and then 3 years later, to year 15. The number of cases detected from the seropositive group (solid bars) declined over time from 20 in the first 2 years, and 12 in the following 2 years, to two in the final 3 years. Detection of NPC cases from the seronegative group (open bars) was delayed for 2 years initially, with only one case being detected during this time. Detection was resumed thereafter, with 8–17 cases, as indicated, being detected in successive periods. As a result of the distinct occurrence of NPC among these two groups of study subjects, prediction by serologic screening declined with time from 95% (20/21) in the first 2 years after enrollment, and 60% (12/20) in the following 2 years, to 12% (2/16) in the final 3 years of follow-up (Figure 2B). Nasopharyngeal carcinoma incidence of the seropositive group (Figure 2C, solid bars) likewise declined from 323 per 10^5^ person-year in the first 2 years through the subsequent periods, to 21 per 10^5^ person-year in the period 13–15 years after enrollment. In contrast, NPC incidence of the seronegative group (solid bars) was markedly lowered to 1.3 per 10^5^ person-year in the first 2 years after screening, rose to 10 per 10^5^ person-year in the following 2 years, and fluctuated between 10 and 21 per 10^5^ person-year in the subsequent periods. Compared with the incidence of the entire study population (hatched bars), the relative risk of NPC in seropositive subjects declined through the successive periods after screening from 12.9 in the first 2 years to 1.7 in years 13–15 (Figure 2D, solid bars), whereas the relative risk of the seronegative subjects was markedly reduced to 0.05 in the first 2 years after screening, rising to 0.4 in the following 2 years thereafter reaching levels similar to the entire study population in subsequent periods.

Building on previous findings (Zeng *et al*, 1983; Chien *et al*, 2001; Ji *et al*, 2003a; Ji *et al*, 2003b), the analysis shows that the seropositive group sustained higher, but declining, levels of NPC risk for 10 years after screening (Figure 2D), a finding attended by a decline in prediction during this period (Figure 2B). On the other hand, the risk sustained by seronegative individuals was markedly lowered in the first 2 years, but rose after 4 years to a level similar to that of both groups of study subjects combined.

### Serologic follow-up of non-NPC subjects

Our study protocol had provided for a regular serologic and clinical follow-up of all seropositive individuals. The follow-up exercise was carried out on seven separate occasions between years 3 and 13 of the study. Seventy-eight per cent of the seropositive subjects complied with the study protocol on the first and second occasions, 72 and 61, 50 and 35, respectively, on the third to the sixth occasion, declining further to 31 and 22% on the final two occasions. Comparable number of subjects from the seronegative group were similarly examined on these occasions. Viral capsid antigen IgA titres in the serum samples taken on these occasions were determined in separate batches. Cutoff titres were set at 1 : 20 or 1 : 40 to achieve a comparable specificity of 90–95% for each sample batch tested as specificity of 92.5% achieved in the initial screening.

**Figure 3:**
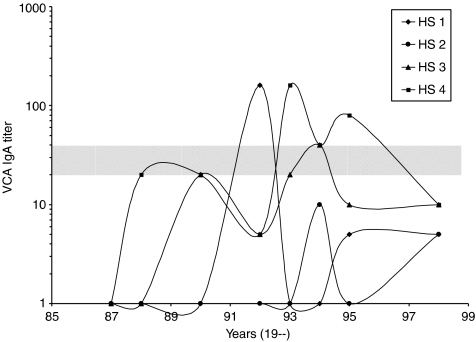
Fluctuating VCA IgA titres among non-NPC subjects during follow-up. Serum samples were taken from four non-NPC subjects (HS 1 to 4) on seven occasions at the indicated times between 1987 and 1998. Cutoff titres were set at 1 : 20 or 1 : 40 for each occasion (shaded zone). Note that antibody titre commonly fluctuated below cutoff level, whereas larger fluctuation, where antibody level rose to and beyond cutoff or declined below cutoff, was less common.

As exemplified in Figure 3, antibody titres of individual non-NPC subjects commonly fluctuated below cutoff level during follow-up, and this is probably owing, to a large extent, to normal assay variation. Larger fluctuation, where antibody levels rose to and beyond cutoff titre, referred hitherto as positive seroconversion, or declined below cutoff, referred hitherto as negative seroconversion, was less common. This type of fluctuation was further analysed as in Table 3 by comparing the percentage of positive and negative seroconversion observed in pairs of consecutive samples taken at different time intervals over the course of the study. Mean frequency of negative seroconversion of 66% for the seronegative group and 57% for the seropositive groups were significantly higher than the corresponding values of positive seroconversion. This suggests that the larger fluctuation in antibody levels observed among non-NPC subjects is due to transient rise of antibody levels, which is most likely induced by EBV reactivation or re-infection. The results further suggest that serologic screening conducted at enrollment may have selected a subpopulation more prone to EBV reactivation, such that mean percentage of seropositive conversion of seropositive group is significantly higher than seronegative group. Mean percentage of negative seroconversion is lower for the seropositive group than the seronegative group, presumably because antibody response to EBV reactivation by the seropositive group might be more vigorous.

### The preclinical EBV serologic profiles of NPC

**Figure 4:**
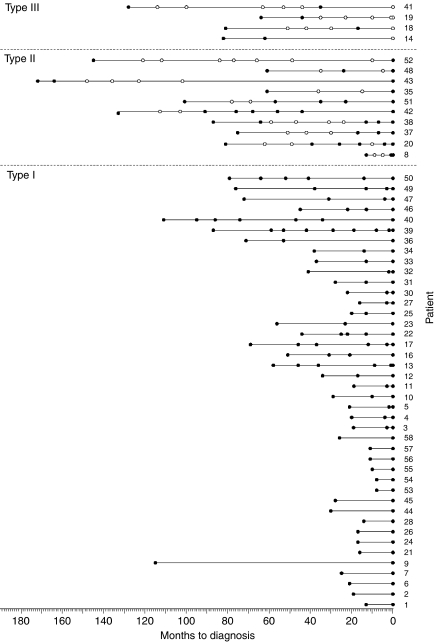
Preclinical EBV serologic profiles of NPC. Fifty-six NPC cases detected from the seropositive group were followed up clinically and serologically for various periods from the time of enrollment to times of diagnosis (month 0). Cases were separated into three categories (types I, II and III) according to their preclinical serologic profiles: serum VCA IgA of type III cases fluctuated between ⩾cutoff titre (closed symbols) and < cutoff titre (open symbols) during follow-up; that of type II cases fluctuated initially and rose to ⩾cutoff titre at or before diagnosis; and that of type I cases were sustained at ⩾cutoff titre throughout follow-up and at diagnosis.

The seropositive cases were followed up 1–7 times before diagnosis, depending on the times of detection and compliance in individual cases, and these include 39 individuals who later developed NPC. Another 16 cases detected from this group were diagnosed on or before the first follow-up exercise. One individual (case 9) was diagnosed 114 months after enrollment, but was not followed up due to non-compliance. Figure 4 depicts VCA IgA serologic changes observed among the 56 NPC cases detected from the seropositive group over the periods 8–172 months before cancer was diagnosed. Among the 39 cases which had been followed up at least once, the results show that 14 became seronegative after enrollment and thereafter antibody levels fluctuated with time, with antibody titre rising to and beyond cutoff (solid circles) and declining below cutoff (open circles), such as seen among non-NPC subjects (see Figure 3). In four of these cases shown, designated ‘type III’, the antibody levels continued to fluctuate and the individuals were seronegative at the time of tumour diagnosis. Another six, designated ‘type II’ cases, were seropositive at diagnosis, having undergone positive seroconversion 1 month (case 8) to 92 months (case 42) earlier. The other four cases (cases 35, 43, 48 and 52) tested seropositive at diagnosis and were presumed to have undergone positive seroconversion earlier, permitting them to be designated as type II cases. The remaining 25 cases followed up were designated ‘type I’ cases. These individuals were seropositive at the time of enrollment and remained so up to the time when the cancer was diagnosed, which was 110 months for case 40. The other 17 cases, all seropositive when diagnosed, were not followed up. Sixteen of these were detected during or before the first follow-up exercise, 8–28 months after enrollment, and in view of the short lapse of time, these individuals were presumed to be type I cases, having sustained high antibody levels in the interim periods, for example cases 56, 57 and 58. Case 9 diagnosed 114 months after enrollment was also deemed type I, although the preclinical serologic status of this case is uncertain.

Taken together, the results served to identify a period immediately preceding the diagnosis of NPC, when serum EBV antibody was raised to and sustained above cutoff titre, although the individuals showed no apparent symptoms of cancer. Based on the time of diagnosis of the 42 type I cases who had entered the window before the initial screening, and 10 type II cases who entered the window after screening, the mean duration of this serologic window was estimated to be 37±28 months. The fluctuating antibody levels observed among the type II cases before this window period and type III cases throughout follow-up may reflect EBV reactivation or re-infection, such as observed among the non-NPC subjects described earlier.

### The relationship between preclinical EBV serologic profiles and NPC tumour progression

**Figure 5:**
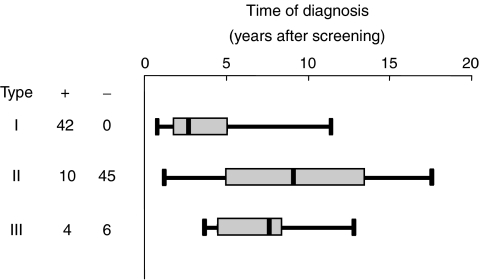
Preclinical serologic profiles and time of subsequent diagnosis of NPC cases. One hundred and seven NPC cases were diagnosed 8 months to 15 years after enrollment; 56 cases were from seropositive group (+) and 51 from seronegative group (−). Cases were separated according to their preclinical serologic profiles into three categories, designated types I, II and to III, as described in Figure 4. Whiskers, boxes and lines in the diagram depict range, interquartile and median times of diagnosis, respectively. Time of diagnosis of the three types of cases is significantly different (*χ*^2^=39.332, df=2, *P*<0.0001).

Serum samples were taken at enrollment, and also at diagnosis, from 51 cases detected from the seronegative group, who presented at OPD. Forty-five of these cases were seropositive at the time of diagnosis, evidently having entered the serologic window similar to the type II cases described above. The other six cases were seronegative at the time of diagnosis, like type III cases. Thus, of a total of 107 cases that were either monitored (39 cases) or could be assessed (68 cases) serologically, Figure 5 (left panel) shows that 97 (90%) had either entered the serologic window (42 type I cases) before screening or would do so at some later times (55 type II cases). Only 10 cases did not undergo a serologic change, possibly due, in part, to the individuality of antibody responses to EBV. Serologic screening served to locate all 42 type I cases in the seropositive group, whereas type II defined most of the cases detected from the seronegative group and type III cases were randomly distributed between the two groups of study subjects.

Detection of cases extended from 8 months to 15 years, and reflected the different stages of preclinical tumour development in individuals when first enrolled into our study. Figure 5, right panel shows that type I cases were at a more advanced stage of preclinical tumour development, and were diagnosed earlier than type II cases. Type I cases were diagnosed 8–114 months after enrollment, with a median interval of 28 months; interquartile intervals were 17 and 51 months, respectively. Type II cases were detected between 13 and 177 months after enrollment, and were at earlier stages of preclinical tumour progression than type I cases. Their median and interquartile intervals for detection were 90 months and 60 and 134 months, respectively, which are much longer than the corresponding values for the type I cases. Type III cases represent a broader spectrum of tumour progression than the other two types of cases, and were detected randomly throughout the study. The median time of detection of these cases was 78 months, and the interquartile intervals were between 45 and 83 months, intermediate between the corresponding values determined for types I and II cases. The time of detection of the three types of cases is significantly different (*χ*^2^=39.332, df=2, *P*<0.0001).

### The relationship between preclinical serologic profiles and NPC risk status

**Figure 6:**
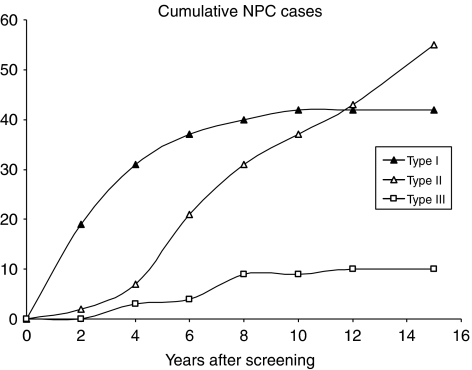
Cumulative detection of NPC cases of different preclinical EBV serologic profiles. One hundred and seven NPC cases were separated into three categories, designated types I–III, according to their preclinical serologic profiles (see Figure 4): type I cases are indicated by closed triangles, type II by open triangles and type III by open squares.

Figure 6 compares the occurrence of NPC cases with different preclinical serologic profiles. Serologic screening identified all 42 type I cases. These account for 19 of all 21 cases detected within the first 2 years from both seropositive and seronegative groups. Type II cases made up most of the cases detected from the seronegative group. Detection of these cases was delayed for about 2 years initially, presumably reflecting the time taken for the cases to transit the serologic window. Detection was resumed at a relatively constant rate thereafter, accompanied by declining detection from among the seropositive subjects. Type III cases were few and randomly detected, and do not contribute to respective risk status of seropositive and seronegative groups. Thus, repeated serologic screening to monitor type II cases as they enter the serologic window can sustain a high prediction rate.

## DISCUSSION

It is a special feature of NPC that patients commonly sustain high levels and a broad spectrum of serum EBV antibodies (Olds *et al*, 1966; Henle *et al*, 1970; Henle and Henle, 1976; Ho *et al*, 1978a; Zeng *et al*, 1983; Cheng *et al*, 2002; Chan *et al*, 2003; Fachiroh *et al*, 2004). The conditions conducive to such a state seem to be attributed not only to the regular presence of the virus in the tumour cells, but also to lymphoid stroma surrounding the tumour (Ho *et al*, 1978b; Niedobitek *et al*, 1996). This serologic feature is also observed among other NPC-like cancers, namely, lymphoepithelioma-like carcinomas, which have the same pathology and, like NPC, regularly harbour EBV (Begin *et al*, 1987; Osato and Imai 1996; Chang *et al*, 2002). Earlier studies suggested that a combination of conditions required to evoke a vigorous antibody response to EBV might be met already in NPC carcinoma *in situ*, that is, at an early stage of disease shortly after a tumour has been initiated (Sam *et al*, 1993; Yeung *et al*, 1993). This contention is generally in line with anecdotal observations that elevation of EBV antibody levels may precede NPC diagnosis by years in some individuals (Ho *et al*, 1978a; Zeng *et al*, 1983; Yip *et al*, 1994).

In the present study, we monitored serologically and clinically 39 cases for different periods of up to 15 years before NPC was diagnosed, and assessed the preclinical serologic status of another 68 cases. The study identified a serologic window marked by a sustained elevation of EBV antibody levels. The mean duration of this window was estimated as 37 months. Among 107 cases, we found that 42 cases, designated type I, had already entered the serologic window at the time of screening, 55 cases, designated type II, would do so at different times later, and only 10 designated type III, did not enter the window, presumably because of the individuality of antibody response. Having already entered the serologic window at screening, the type I cases are at more advanced stages of preclinical tumour development and hence are detected earlier than the type II cases, who would enter the serologic window at later times. The type I cases became clinically manifested after a delay of 8–114 months after screening. The median interval for detection of type I cases was 28 months and interquartile values were 17 and 51, respectively. Detection of the type II cases was further delayed by 13–177 months; the median interval was 90 months and interquartile values were 60 and 134, respectively, which are much longer than the corresponding values for type I cases. Type III cases were detected randomly throughout follow-up, and the median and interquartile intervals for detection of these cases were intermediate between the corresponding values for type I and II cases. It was concluded from these findings that the serologic window is a common event in the preclinical phase of NPC development, with 97 (90%) of 107 cases either having entered the serologic window before screening or doing so after, thus confirming the contention that the conditions required to evoke a vigorous antibody response to EBV are met before the cancer becomes clinically manifested.

Serologic screening takes advantage of the above-described features of preclinical tumour development to predict NPC. Our effort located all 42 type I cases among the seropositive subjects, and predicted 19 of 21 cases detected from the entire study population within 2 years of screening. Relative NPC risk of the seropositive subjects was also highest during this period. The prediction rate and risk level declined at later times in proportion, with the declining number of type I cases detected. Cases detected from the seronegative groups were mainly made up of type II cases, which are at earlier stages of preclinical tumour development than type I cases. It was observed that the detection of type II cases was delayed for about 2 years initially, presumably reflecting the time taken for the cases to traverse the serologic window. Thereafter, cases were detected at a relatively constant rate. These findings show that repeated serologic screening of a target population at regular intervals may serve as a sentinel to monitor type II cases as they enter the serologic window, and provide a valuable high prediction rate for these individuals.

Although cure can still be achieved in all stages of NPC in the absence of distant metastases, the prognosis is significantly worse in advanced-stage disease. Patients with early-stage disease have not only improved survival rate, but also reduced toxicities of treatment (Chan *et al*, 2004). Early disease symptoms are innocuous, however, and even agreed participation in a program, such as ours, was not accompanied by an enhanced awareness of the cancer by the study subjects themselves, or improved early detection. Consequently, 80% of our study cases that were diagnosed by clinical symptoms and 79% of the concurrent control cases of newly diagnosed NPC during the study period, were diagnosed with advanced-stage disease. Confirming previous reports (Zeng *et al*, 1983; Ji *et al*, 2003b), we demonstrated in a large-scale study that this trend could be reversed using a screening program that employed serological testing and clinical examination. Here, 68% of the cases thus detected by the screening program were diagnosed at the early stage I or II disease.

Nasopharyngeal carcinoma mainly affects adults in their most productive age, between 35 and 65 years (Yu and Yuan, 2002). Our findings suggest that effective control of this cancer can be achieved by repeating serologic screening every 2 years to identify cases as they enter the serologic window. We estimate that such a program would sustain a prediction rate of 90% and more importantly about two-third of these cases diagnosed by the program belongs to early-stage disease. Apart from benefits to the afflicted individuals, such a program, when implemented community-wide in high-incidence areas for this cancer, would have important economic and sociological ramifications. We believe that we have shown this to be a practical proposition. Moreover, the VCA IgA assay used in this study, the only suitable assay at the time, has now been replaced by high-throughput objective assays produced with purified recombinant EBV proteins, which afford greater specificity and sensitivity than traditional assays, especially when they are used in combination (Cheng *et al*, 2002). Detection of the EBV gene in nasopharyngeal swabs from symptomatic patients has been shown to be highly predictive of symptomatic NPC (Hao *et al*, 2003; Tsang *et al*, 2003), and the incorporation of this approach in routine NPC screening of apparently healthy subjects is expected to enhance early detection of the cancer.

## Figures and Tables

**Table 1:** NPC cases detected

	**VCA IgA status at enrollment^a^**		
	**Seropositive**	**Seronegative**	**Total**
Number of subjects enrolled	3093	38 955	42 048
NPC cases detected at enrollment	39	1	40

Follow-up examinations^b^	13186	9093	22 279
*Number of NPC cases detected*			
By routine screening^c^	34	0	34
In study clinic after onset of symptoms^d^	22	51	73
In other hospital after onset of symptoms^e^	0	24	24

NPC case detected after enrollment	56	75	131
Incidence (cases/10^5^ person-year)^f^	120.70	12.84	20.77
Relative risk^g^	5.81	0.62	1.00
			

NPC=nasopharyngeal carcinoma; VCA.IgA=viral capsid antigen IgA.

Study subjects were serologically screened and given a clinical examination at the time of enrollment.

a Serum EBV VCA IgA titre ⩾1:5 designated seropositive, <1:5 designated seronegative.

b Performed on eight occasions over the subsequent 13 years either.

c By clinical examination conducted on these occasions.

d when symptomatically presented to the clinic of our hospital.

e Traced to other centers up to 15 years after enrollment.

f Calculated over 15 years.

g Calculated as ratios of cumulative incidence of seropositive or seronegative groups to both groups combined.

All NPC cases were WHO class II or III non-keratinizing carcinoma, except for five WHO class I cases of keratinized carcinoma.

**Table 2:** Clinical status of NPC cases detected

		**AJCC/UICC stage**				
**Detection**	**Number of NPC cases**	**I**	**II**	**III**	**IV**	**% I and II**
Routine screening:	74	44	6	16	8	67.6
After onset of symptoms	97	7	12	38	40	19.6
New cases referred to our clinic	1629	173	164	578	714	20.7

AJCC/UICC=American Joint Committee of Cancer/International Union Against Cancer; NPC=nasopharyngeal carcinoma.

One hundred and seventy-one NPC cases were detected from study subjects either by clinical examination during routine screening, or when patients presented with symptoms of NPC (see Table 1 for details). Newly diagnosed cases, usually with presence of symptoms and referred to our out-patients clinics during the same time, served as concurrent control cases. Percentage of stages I and II cases detected by routine screening is significantly higher than that detected after onset of symptoms (*χ*^2^=154.88, *P*=0.000), and that of the latter is similar as new cases referred to our clinics (*χ*^2=^1.984, *P*=0.576).

**Table 3:** Fluctuating VCA IgA titres of non-NPC subjects during follow-up

	**Negative seroconversion**				**Positive seroconversion**			
	**Seropositive group**		**Seronegative group**		**Seropositive group**		**Seronegative group**	
**Years**	***n***	**%**	***n***	**%**	***n***	**%**	***n***	**%**
1987–88	1705	65	0		0		1153	12
1988–90	489	70	44	89	1025	15	376	5
1990–92	374	45	23	65	1946	23	557	8
1992–93	393	47	55	67	876	18	728	5
1993–94	382	24	64	44	1000	24	750	7
1994–95	387	53	70	67	440	17	546	5
1995–98	133	68	27	59	401	14	344	6
1998–99	74	66	30	80	362	7	312	4
Mean±s.d.	3937	57±17	313	66±14	6050	19±4	4766	7±3

NPC=nasopharyngeal carcinoma; s.d.=standard deviation; VCA IgA=viral capsid antigen IgA.

Pairs of consecutive serum samples (*n*) were taken at indicated intervals during follow-up from non-NPC subjects, who were initially screened at the time of enrollment as having ⩾cutoff VCA IgA titre (seropositive group) or < cutoff VCA IgA titre (seronegative group). Fluctuating antibody level was indicated, when antibody levels rose to or beyond cutoff titre (positive seroconversion) or declined below cutoff titre (negative seroconversion). Pairs of consecutive serum samples (*n*) were taken at indicated intervals during follow-up from non-NPC subjects, who were initially screened at the time of enrollment as having ⩾cutoff VCA IgA titre (seropositive group) or < cutoff VCA IgA titre (seronegative group). Mean percentage of negative seroconversion of either seropositive or seronegative group is significantly higher than the corresponding values of mean percentage of positive seroconversion (*χ*^2=^1536.33, *P*<0.001; *χ*^2^=1026.66, *P*<0.001); mean percentage of positive seroconversion of the positive group is significantly higher than that of the seronegative group (*χ*^2=^299.33, *P*<0.001); and mean percentage of negative seroconversion is significantly higher for the seronegative group than seropositive group (*χ*^2^=13.82, *P*<0.01).
